# Forefoot pathology in rheumatoid arthritis identified with ultrasound may not localise to areas of highest pressure: cohort observations at baseline and twelve months

**DOI:** 10.1186/1757-1146-4-25

**Published:** 2011-11-23

**Authors:** Catherine J Bowen, David Culliford, Ruth Allen, James Beacroft, Anita Gay, Lindsey Hooper, Jane Burridge, Christopher J Edwards, Nigel K Arden

**Affiliations:** 1 Faculty of Health Sciences, University of Southampton, Southampton, UK; 2 Musculoskeletal Biomedical Research Unit, University of Oxford, Oxford, UK; 3 Faculty of Medicine, University of Southampton, Southampton, UK; 4 Department of Rheumatology, Southampton University Hospitals NHS Trust, Southampton, UK; 5 Wellcome Trust Clinical Research Facility, Southampton University Hospitals Trust, Southampton, UK; 6 MRC Epidemiology Resource Centre, University of Southampton, Southampton, UK

## Abstract

**Background:**

Plantar pressures are commonly used as clinical measures, especially to determine optimum foot orthotic design. In rheumatoid arthritis (RA) high plantar foot pressures have been linked to metatarsophalangeal (MTP) joint radiological erosion scores. However, the sensitivity of foot pressure measurement to soft tissue pathology within the foot is unknown. The aim of this study was to observe plantar foot pressures and forefoot soft tissue pathology in patients who have RA.

**Methods:**

A total of 114 patients with established RA (1987 ACR criteria) and 50 healthy volunteers were assessed at baseline. All RA participants returned for reassessment at twelve months. Interface foot-shoe plantar pressures were recorded using an F-Scan^® ^system. The presence of forefoot soft tissue pathology was assessed using a DIASUS musculoskeletal ultrasound (US) system. Chi-square analyses and independent t-tests were used to determine statistical differences between baseline and twelve months. Pearson's correlation coefficient was used to determine interrelationships between soft tissue pathology and foot pressures.

**Results:**

At baseline, RA patients had a significantly higher peak foot pressures compared to healthy participants and peak pressures were located in the medial aspect of the forefoot in both groups. In contrast, RA participants had US detectable soft tissue pathology in the lateral aspect of the forefoot. Analysis of person specific data suggests that there are considerable variations over time with more than half the RA cohort having unstable presence of US detectable forefoot soft tissue pathology. Findings also indicated that, over time, changes in US detectable soft tissue pathology are out of phase with changes in foot-shoe interface pressures both temporally and spatially.

**Conclusions:**

We found that US detectable forefoot soft tissue pathology may be unrelated to peak forefoot pressures and suggest that patients with RA may biomechanically adapt to soft tissue forefoot pathology. In addition, we have observed that, in patients with RA, interface foot-shoe pressures and the presence of US detectable forefoot pathology may vary substantially over time. This has implications for clinical strategies that aim to offload peak plantar pressures.

## Background

Patients with rheumatoid arthritis (RA) present with pain, changes in gait, foot deformity and restrictions in the choice of footwear [1-4]. This has led to the development of guidelines for the assessment and management of foot complications associated with RA. Annual foot health screening is recommended with the aim of identifying changes in foot health and monitoring foot health interventions [5,6]. However, in a recent study of patients with RA we demonstrated that a high percentage of soft tissue pathology within the forefoot detectable by musculoskeletal ultrasound (US) was often missed by clinical examination [7]. In addition, we found that US detectable soft tissue pathology within the forefoot was clinically relevant but varied in prevalence over time and hypothesised that this was not necessarily due to RA disease but potentially associated with mechanical factors [8].

Measurement of foot-shoe interface pressures is increasingly used in clinical practice to determine clinical interventions, such as foot orthoses, for patients with RA, yet there is very little evidence for this practice over time. In cross-sectional studies peak plantar pressures are most often reported and evidence shows the forefoot as the region with the highest peak plantar pressures [9-14]. Notably, the clinical relations of plantar pressures in RA patients are less well understood. Some have attempted to address this using radiographic erosion scores that show associations of MTP joint erosions with peak plantar foot pressures [11,13,15]. A main criticism of the radiological erosion scores is that they only give information on prevalent joint damage and are insensitive to RA soft tissue changes [16,17]. We therefore decided to investigate patterns of foot-shoe interface pressures and presence of US detectable soft tissue pathology in a cohort of RA participants at two time points, baseline and twelve months.

## Methods

The optimal research design was considered to be a longitudinal cohort study in which the foot pathology and foot pressure characteristics of a heterogenous group of patients who have RA were assessed at two time points. The use of two cross sectional time points within the same population allows for better understanding of the effect of variability in pathophysiology of RA within the foot over time. Embedded within the design of study was a case reference study, to enable comparisons of baseline demographic and clinical characteristics of the RA study sample with healthy control participants.

Approval for the study was obtained from the Southampton and South West Hampshire research ethics committee for the RA participants and the Faculty of Medicine, Health and Life Sciences, University of Southampton Research ethics committee for the healthy participants. All participants gave informed written consent prior to participation.

### Study population

The study population consisted of a consecutive sample of 114 RA patients who attended the Rheumatology Department at Southampton General Hospital. Data collection took place in the Wellcome Trust Clinical Research Facility, Southampton General Hospital, between August 2006 and December 2008. These individuals were participants in the RA Feet Ultrasound project (FeeTURA), a prospective cohort study designed to investigate the epidemiology of forefoot pathology in RA patients. The point of entry into FeeTURA included all patients who have RA who were attending for routine rheumatological clinical care during the recruitment period (April 2006 - April 2007). Previous publications have described the high prevalence of forefoot bursal hypertrophy in this patient group [7,8]. The present analysis was conducted to examine foot pressure outcomes in a subgroup of the FeeTURA project participants.

To be eligible for participation in the parent FeeTURA study, participants had to be over the age of eighteen and have a positive diagnosis of RA as defined by the previous American College of Rheumatology (ACR) 1987 criteria [18]. Patients were excluded from the study if they had a history of previous forefoot surgery, received a corticosteroid injection to the forefoot within the three months prior to this study, had an additional musculoskeletal disease (e.g. primary osteoarthritis, gout, Paget's, systemic lupus erythematosus), or had a serious medical (other than RA) or psychological disorder that would prevent completion of the study protocol. Also, for this foot pressure study, individuals who could not walk five metres were excluded.

A total of 149 patients were recruited into the parent FeeTURA study and assessed at baseline (start of the study). The number dropped to 120 who were re-assessed at twelve months due to non-responses (n = 21), death (n = 1), illness (n = 6) and non-eligibility based on an inability to walk five metres (n = 1). During the pre-selection process for this investigation, data from a further 6 subjects that were mal-recorded at either baseline or twelve months were excluded from the final analyses.

A gender matched healthy comparison group was recruited from the students and staff of the University of Southampton and assessed at the start of the study at baseline only. The inclusion criteria were an age of 18 + years, no positive diagnosis of an inflammatory arthropathy and all participants had to fulfill the same exclusion criteria as those for the RA group. Fifty healthy participants (37 female, 12 male; mean age 33.2 years, range 19-61; mean weight 74 kg, range 54.5-120) were recruited and plantar pressure measurements and ultrasound data subsequently recorded. Participants were instructed to attend the visit wearing comfortable flat shoes that they wore the most at the time.

### Assessment of demographic and clinical characteristics of the RA participants

Demographic data including age, gender, weight, height, disease duration and presence of rheumatoid factor was recorded. Information regarding current medication including Disease Modifying Anti-Rheumatic Drug (DMARD) use was obtained from the patients' clinical notes. C-reactive protein (CRP) and Erythrocyte Sedimentation Rate (ESR) values were obtained from the clinical/laboratory database. Clinical activity of RA disease was assessed by the disease activity score 28 tender and swollen joint count (DAS28-ESR) [19] and was obtained from the patients' clinical notes within one month of the visit.

All foot assessments were conducted by a single investigator (CB) at both time points and followed recommended guidelines for clinical assessment [5,6]. This included observation of the presence of foot deformities: hallux abducto valgus (HAV), 5^th ^metatarsophalangeal (MTP) joint exostosis, lesser toe deformity, MTP joint subluxation, pes cavus and pes planus. Motion at the ankle, sub-talar, mid-tarsal and first MTP joints were assessed and classified as full motion, limited motion or rigid according to clinical guidelines [5,6]. Information regarding use of foot orthotic devices, presence of foot ulceration and access to clinical foot services was also recorded.

Footwear was assessed and categorised as either prescribed therapeutic footwear or retail (shop bought) footwear. Footwear was further noted as being suitable or not suitable according to fit and style (e.g. court styles and high heel/stiletto shoes were deemed unsuitable). Due to the high numbers of participants and the highly emotive factors associated with both prescribed therapeutic and retail footwear [4,20,21] it was neither economically feasible nor clinically desirable to standardise footwear between visits. Participants were instructed to attend each visit wearing comfortable flat shoes that they wore the most at the time.

Both subscales of the Leeds Foot Impact Scale Questionnaire (LFIS), impairment/footwear (LFIS_IF_) and activity limitation/participation restriction (LFIS_AP_) previously validated for use in RA populations [22] were used to identify patient reported foot impact. LFIS_IF _contains twenty one items related to foot pain and joint stiffness, as well as footwear related impairments with a total score range 0 -21. LFIS_AP _contains thirty items related to activity limitation and participation restriction with a total score range 0-30 [22]. Responses to each question are dichotomized as yes or no and scoring is a simple tally for each domain [22] with 4 or less suggested to represent good foot health and scores higher than 4 representing poor foot health [23].

### Foot pressure measurement

A portable pressure measurement device, the FScan^® ^in-shoe system, (Tekscan Inc. USA) was used to record foot-shoe interface pressures. The FScan^® ^system has recently been demonstrated as highly reliable and suitable for measurement of plantar foot pressures in RA patients in clinical practice [24]. The system is calibrated to weight and uses Force Sensing Resistor (FSR) technology to enable dynamic, real time measurement to measure the interface between the foot and footwear. The instrumented insole is composed of 960 Sensing Elements/Foot (Sensels), each 0.15 mm in thickness with a density of four sensors per cm^2^. It was trimmed to fit footwear so that it did not interfere with either walking, comfort or fit of footwear (FScan^® ^system features, Tekscan US).

A predetermined walkway of approximately five metres was established along the length of the clinical room. Each participant was initially asked to walk the length of the walkway to familiarise themselves with the protocol and become accustomed to the cables. All participants were asked to walk with their own footwear in a straight line at a comfortable walking speed, away from the FScan^® ^system so that any cable trip hazard was avoided. An identical standard recommended protocol was followed for each participant to minimise variations in recordings.

The data acquisition parameters were prescribed to record 10 seconds of information with a 165Hz sampling frequency. The FScan^® ^system automatically records the individual data from all of the sensors and estimates the pressure distribution on the plantar aspect of the feet during each footstep, storing data on the system for later analysis.

F-Scan^® ^sensors are marketed as re-useable and previous laboratory work has identified sensor life-spans of 40 trials over ten metres [25]. However, we are aware of reported limitations of the FScan^® ^pressure measurement system employed, especially the reliability of the sensors has been questioned [9,26]. Therefore, we conducted a repeated measures same subject study to test reliability of the sensors for clinical use. Our findings suggested that there was a trend in the loss of FScan^® ^sensor lifespan following multiple clinical uses after 20 trials. To minimise the variation and inaccuracy of data recordings we adopted a strict protocol as follows:

i. The sensors were placed within the participants' footwear with the backing intact to minimise damage as recommended [25].

ii. Each time a sensor in our study was used the participant code, number of 'walks'and number of steps taken was noted on the sensor log sheet.

iii. Sensors were discarded after maximum use of 20 times (over five metres) or if physical damage to the sensor was observed.

iv. In addition, with careful recalibration of sensors at each trial and observation of the walking trials, any mal-recordings were identified and excluded from the final data analysis.

The FScan^® ^system was set to automatically discard the first and last footsteps. The third footstep was selected for analysis as this was considered representative of mid-gait and peak pressures were calculated. Using the FScan^® ^standard masking software, the footprints were divided into six segments, A (lateral-forefoot, ie. 3^rd ^to 5^th ^MTP joints), B (medial-forefoot, ie.1^st ^to 2^nd ^MTP joints), C (lateral-midfoot), D (medial-midfoot), E (lateral-rearfoot), F (medial-rearfoot). The location (ie. segment A, B, C, D, E or F) was noted in which the peak pressure of the footstep was identified.

To determine relations in locations of US detectable forefoot pathology and location of peak pressure, cases in which the peak pressure was displayed within the forefoot as either medially dominant (segment B) or laterally dominant (segment A) were selected for analysis.

### Ultrasound assessments

All US scans were performed immediately after the clinical foot examinations and foot pressure measurements by a single investigator (CB). We attempted to reduce the effect of investigator bias by maintaining a systematic order to the data collection and using experienced independent data handlers to double enter and clean all the information onto the data sheet.

A Diasus ultrasound system (Dynamic Imaging Ltd, UK) was used to image the forefoot of both feet to determine the presence of forefoot pathology (MTP joint synovial hypertrophy and erosion and plantar forefoot bursal hypertrophy). The Diasus ultrasound system (Dynamic Imaging Ltd. Scotland UK) operates as a system with dual probe of which we employed the 8-16 MHz, footprint 26 mm, for dorsal scans and the 5-12 MHz linear probe, footprint 40 mm, for plantar scans. Scanning was in B-Mode and recorded according to standard guidelines for MTP joint pathology [27] and previous recommendations for detection of plantar forefoot bursal hypertrophy [28]. Good image acquisition and interpretation agreement (kappa 0.702; p < 0.01) with an expert US radiologist was confirmed prior to data collection [28].

**Figure 1:**
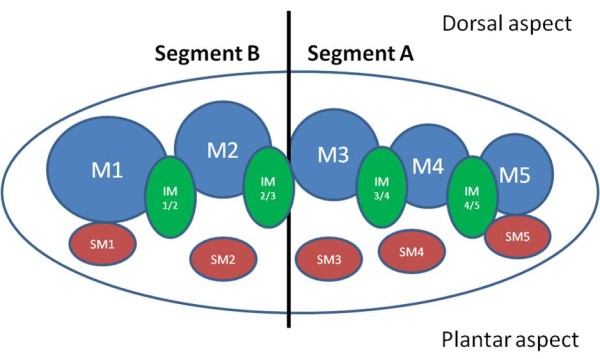
**Diagramatic representation of the division of the forefoot into medial and lateral pathology**. Legend: M = metatarsal head; IM = intermetatarsal space; SM = sub-metatarsal area.

The presence or absence of MTP joint synovial hypertrophy and erosion was recorded in the first to fifth MTP Joints. The presence or absence of forefoot bursal hypertrophy was recorded in the intermetatarsal (IM) spaces 1/2, 2/3, 3/4, 4/5 and the sub-metatarsal (SM) head areas 1 - 5. Locations of forefoot pathology were allocated as A (lateral-forefoot, ie. 3^rd ^to 5^th ^MTP joints including IM spaces 3/4 and 4/5, SM areas 3,4,5) or B (medial-forefoot, ie.1^st ^to 2^nd ^MTP joints, including IM spaces 1/2 and 2/3, SM areas 1,2) (Figure 1).

To facilitate analysis for associations, the scores for US detectable pathology presence were summated as follows:

Segment A (Lateral): presence of MTP joint synovial hypertrophy 3, 4 and 5 + presence of MTP joint erosion 3, 4 and 5 + presence of forefoot bursal hypertrophy IM 3/4, 4/5, SM 3, 4, 5. Segment B (Medial): presence of MTP joint synovial hypertrophy 1, 2 + presence of MTP joint erosion 1, 2 + presence of forefoot bursal hypertrophy IM 1/2, 2/3, SM 1, 2.

### Analysis

Using prior data [10] for normally distributed matched pairs with peak pressure as the primary outcome, power calculations indicated that the sample size of 114 for 90% power was more than adequate to detect differences in outcomes of pathology and peak plantar pressures. All data analyses were conducted using Statistical Package for the Social Sciences (SPSS) version 17.0 software (SPSS, Chicago IL). Unless otherwise noted, a p value of less than 0.05 was considered the critical level to determine statistical significance.

The analyses mainly focused on descriptive changes in the presence and location of US detectable forefoot pathology and value and location of peak pressure at baseline for both RA and healthy participants and after a period of twelve months for RA participants only. Demographic and clinical characteristic information is presented as mean and standard deviations (+/-SD). The foot specific characteristics of the study participants are presented as frequencies of occurrence and graphically as bar charts. Chi-square (χ^2^) analyses were used to determine differences within location of peak pressures between RA and healthy participants at baseline and for RA participants with location of peak pressures and location of forefoot pathology from baseline to 12 months. Chi-square (χ^2^) analyses were also used to determine differences in peak pressure values and locations according to footwear type of the RA participants at baseline and twelve months.

Independent sample t-tests were used to determine differences between peak pressure values for RA and control participants and paired t-tests were used to determine differences for peak pressure values for the RA participants from baseline to twelve months. Change in the demographic and clinical variables was calculated as person specific data and is presented as frequencies.

Pearson's correlation coefficient was used to determine interrelationships between the US detectable pathology and values of peak pressure within each forefoot segment at baseline and at 12 months, as well as between the changes in forefoot pathology and changes in peak pressure values after 12 months. The distribution of data for both peak pressures and US detectable forefoot pathology were found to be approximately normal, justifying the use of a parametric method for assessing correlation.

## Results

### RA participant demographics

One hundred and fourteen patients (93 female and 21 male; 22 seronegative, 89 seropositive, 3 missing data) were included the study. The mean age of the RA participants was 59.6 years (SD:12.0; range: 25-87) and mean disease duration was 11.8 years (SD: 10.3; range 0.6-43) at baseline start of the study. The group of RA participants were heterogeneous as can be seen by the clinical and demographic variables for baseline and twelve months (Table 1). Analysis of group means showed no significant change over the 12 month period for all variables. However when person specific data was calculated it is notable that change had taken place over the twelve month period with almost equal numbers of participants increasing as decreasing for each variable.

**Table 1:** Demographic and clinical characteristics of the RA participants at baseline and 12 months (N = 114).

	Baseline Mean (± SD)	12 months Mean (± SD)	Raw Change Mean (± SD)	PSC Up	PSC Down	PSC No Change
Weight (kg)	73.3 (15.5)	73.2 (15.4)	- 0.1 (4.3)	56 (49%)	57 (50%)	1 (1%)

Wellbeing (VAS)	40.1 (23.8)	36.2 (22.8)	- 3.3 (25.8)	56 (49%)	56 (49%)	2 (2%)

ESR (mm/hour)	22.9 (18.6)	24.6 (20.3)	1.8 (16.9)	*47 (41%)	53 (47%)	6 (5%)

CRP (mg/litre)	12.4 (19.5)	14.8 (25.0)	2.9 (29.1)	*50 (44%)	39 (34%)	12 (10%)

DAS-28	3.9 (1.3)	4.1 (1.4)	0.2 (1.8)	*35 (31%)	25 (22%)	17 (15%)

LFIS_IF _(x/21)	10.6 (4.9)	10.3 (4.8)	- 0.4 (3.3)	44 (39%)	53 (47%)	17 (15%)

LFIS_AP _(x/30)	16.5 (9.5)	16.6 (9.7)	- 0.1 (5.7)	*53 (47%)	43 (38%)	17 (15%)

Key:*ESR 8 missing data; CRP 13 missing data; DAS-28 37 missing data; LFIS_AP _1 missing data.

Legend: PSC = person specific change n(%); VAS = visual analog score; ESR = Erythrocyte sedimentation rate; CRP = C-reactive proteins; DAS-28 = 28 joint disease activity score; LFIS_IF _= Leeds Foot Impact Score, impairment/footwear subscale; LFIS_AP _= Leeds Foot Impact Score, activity participation limitation subscale.

Pharmacological treatment appeared to be stable within the group. At baseline the participants' regular treatment of RA included 66% (n = 75) taking methotrexate and 47% (n = 53) taking anti-TNFα (Adalimumab, Infliximab, Etanercept) therapy. At 12 months the participants' regular treatment of RA included 71% (n = 81) taking methotrexate and 46% (n = 52) taking anti-TNFα (Adalimumab, Infliximab, Etanercept) therapy.

### RA participant clinical foot characteristics

Patient reported foot impact appears high with mean impairment/footwear scores of 10.6/21 (baseline) and 10.3/21 (twelve months) and mean activity limitation/participation restriction 16.5/30 (baseline) and 16.6/30 (twelve months) (Table 1). Analysis of person specific data shows that both scores changed over the twelve month period with almost equal numbers of participants experiencing an increase in foot impact as those experiencing a decrease (Table 1).

**Figure 2:**
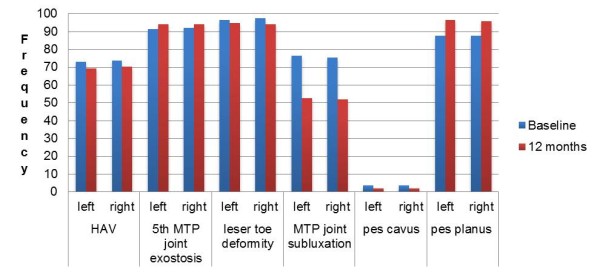
**Clinical foot characteristics of the RA participants at baseline and 12 months**.

**Figure 3:**
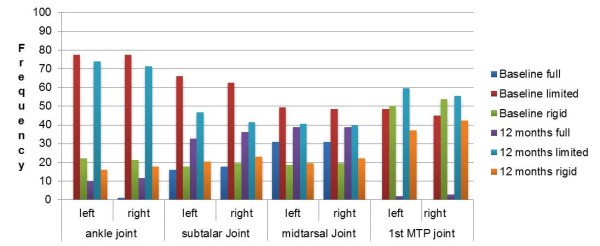
**Foot joint characteristics of the RA participants at baseline and 12 months**.

A high percentage of symmetrical foot deformity was observed for HAV, 5^th ^MTP joint exostoses, lesser toe deformities, MTP joints 1-5 subluxation and pes plano valgus foot position at both baseline and 12 months (Figure 2). From Figure 3, the highest proportion of participants had limited ranges of motion in their foot joints at both time points.

At baseline 56% (n = 64) had recorded foot symptoms in their clinical notes, 61% (n = 70) had seen a chiropodist or podiatrist but only 31% (n = 35) were currently receiving clinical foot care on a regular basis. No participants had recorded presence of foot ulceration although 7% (n = 8) reported a previous history of foot ulceration. Access to clinical foot care appeared to have improved slightly at 12 months with 42% (n = 48) receiving clinical foot care on a regular basis. Foot ulceration was noted in 2% (n = 2) at twelve months.

At baseline 97% (n = 111) wore retail shoes and 3% (n = 3) wore prescribed therapeutic shoes. Of the retail shoes, 16% (n = 18) were deemed unsuitable by the podiatrist. At 12 months 93% (n = 106) wore retail shoes and 6% (n = 7) wore prescribed therapeutic shoes. Of the retail shoes, 6% (n = 7) were deemed unsuitable by the podiatrist. At baseline 11% (n = 12) wore simple insoles, 4% (n = 5) wore moulded insole devices and 3% (n = 3) wore total contact foot orthoses. Slightly more wore simple insoles (15%, n = 17), moulded insole devices (6%, n = 7) and total contact foot orthoses (4%, n = 5) at twelve months.

### Foot pressure characteristics (RA participants and healthy controls)

Peak pressure values within the whole footstep were significantly different at baseline between the RA participants and the group of healthy control participants (Table 2). To assess whether these differences could be due to the confounding influences of age and weight, an analysis of variance (ANOVA) was performed. After adjustment for age and weight the results remained significant (p < 0.001).

**Table 2:** Foot pressure characteristics of the RA participants compared to the control group at baseline.

	RA Mean (SD)	Control Mean (SD)	Students t-test (95% CIs)
Left peak pressure (kPa)*	559.1 (281.6)	460.9 (146.0)	t = 2.330, p = 0.021 (14.96-181.45)

Right peak pressure (kPa)*	581.5 (298.0)	449.5 (167.8)	t = 2.931, p = 0.004 (43.0-220.9)

Key: *Denotes significance at p < 0.05

No significant differences were found in peak pressure values for the RA participants from baseline to 12 months (Table 3). However when person specific data was calculated it is notable that change had taken place over the twelve month period with almost equal numbers of participants having an increase (left 47%, right 47%) in peak foot pressure as those who had a decrease (left 53%, right 54%).

**Table 3:** Foot pressure characteristics of the RA participants at baseline and 12 months.

	Baseline Mean (SD)	12 months Mean (SD)	Students t-test (95% CIs)	PSC increase	PSC decrease
Left peak pressure (kPa)	559.1 (281.6)	565.1 (291.3)	t = -0.180, p = 0.857 (-72.6-60.5)	54 (47%)	60 (53%)

Right peak pressure (kPa)	581.5 (298.0)	582.3 (396.7)	t = -0.020, p = 0.984 (-84.7-83.0)	53 (47%)	61 (54%)

Legend: PSC = person specific change n(%)

**Figure 4:**
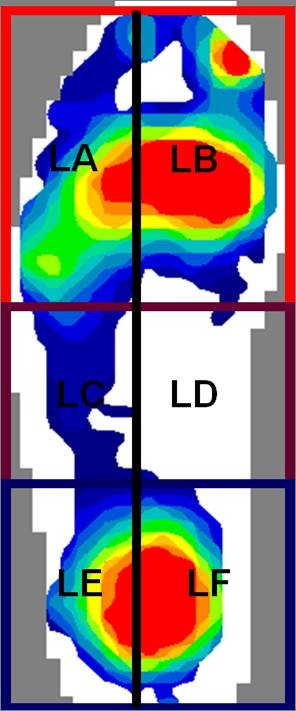
**The most common pattern of foot pressure seen in one RA participant's left foot pressure-map**. Legend: LA = left lateral forefoot segment; LB = left medial forefoot segment; LC = left midfoot lateral segment; LD = left medial midfoot segment; LE = left lateral rearfoot segment; LF = left medial rearfoot segment.

Once the footprint was segmented into the six components, the majority of RA participants displayed peak pressure values within the forefoot region (ie. segments A and B) in both feet (Table 4 Figure 4). At baseline no significant differences were found in the locations of the peak pressures between the RA participants and the group of healthy control participants (left χ^2 ^= 0.185, df = 1, p = 0.185 or right feet χ^2 ^= 0.004, df = 1, p = 0.947).

**Table 4:** Location of forefoot pressures in the RA participants(n (%)) at baseline and twelve months.

		Forefoot		Midfoot		Rearfoot	
		**Lateral** **A**	**Medial** **B**	**Lateral** **C**	**Medial** **D**	**Lateral** **E**	**Medial** **F**

Baseline							

	Left	37 (33%)	63 (55%)	0	0	2 (2%)	12 (10%)
	Right	20 (18%)	70 (61%)	2 (2%)	1 (1%)	5 (4%)	16 (14%)

Twelve Months							

	Left	37 (33%)	56 (49%)	1 (1%)	0	7 (6%)	13 (11%)
	Right	24 (21%)	72 (63%)	1 (1%)	0	4 (4%)	13 (11%)

When the locations of peak pressures were analysed for the RA participants at baseline and twelve months significant differences were found for both left (χ^2 ^= 12.063, df = 1, p = 0.001) and right feet (χ^2 ^= 4.627, df = 1, p = 0.031). Further analysis of person specific data showed that peak pressure location was stable in only 61% (n = 69) of participants in the right foot and only 34% (n = 39) in the left foot (Table 5). This suggests that, in RA patients, whilst the location of peak foot pressures is predominantly within the medial aspect of the forefoot, this may change over time.

**Table 5:** The presence of person specific(n) stable peak pressure location over 12 months for the RA participants.

Peak pressure location	Left	Right
Stable presence lateral A	21	7

Stable presence medial B	13	54

Stable presence C,D,E,F	5	8

Total stable peak pressure	39	69

Percentage of cohort	34%	61%

### US detectable forefoot pathology (RA participants)

Frequency of the presence of US detectable forefoot pathology was high. When categorized as medial (segment B) or lateral (segment A), the presence of MTP joint erosions appeared to be predominantly lateral. MTP joint synovial hypertrophy appeared to be predominantly lateral at baseline, but was different at twelve months being predominantly medial. Plantar forefoot bursal hypertrophy has been reported previously in the parent FeeTURA study [7,8] and in this sub analysis was also predominantly lateral at both baseline and twelve months (Table 6**)**.

**Table 6:** Location of forefoot pathology in the RA participants(n (%)) at baseline and twelve months.

Pathology		Lateral dominance	Medial dominance	Equally distributed	No pathology
Baseline					

SynHy	Left	37 (33%)	19 (17%)	15 (13%)	43 (38%)
	Right	31 (27%)	25 (22%)	10 (9%)	48 (42%)

Erosion	Left	94 (83%)	1 (1%)	6 (5%)	11 (10%)
	Right	88 (77%)	2 (2%)	6 (5%)	18 (16%)

BurHy	Left	58 (51%)	18 (16%)	20 (18%)	18 (16%)
	Right	56 (49%)	13 (11%)	27 (24%)	18 (16%)

Twelve Months					

SynHy	Left	15 (13%)	34 (30%)	7 (6%)	58 (51%)
	Right	19 (17%)	26 (23%)	14 (12%)	55 (48%)

Erosion	Left	96 (84%)	1 (1%)	11 (10%)	5 (4%)
	Right	89 (78%)	1 (1%)	14 (12%)	10 (9%)

BurHy	Left	61 (54%)	7(6%)	32 (28%)	14 (12%)
	Right	63 (55%)	14 (12%)	22 (19%)	15 (13%)

Legend: SynHy: MTP joint synovial hypertrophy; BurHy: Forefoot bursal hypertrophy.

When person specific changes were analysed, MTP joint erosion accounted for the least change in status and location and thus the most stable pathology whilst just under half of participants were observed to have a stable status and location of MTP joint synovial hypertrophy and forefoot bursal hypertrophy (Table 7). This suggests that the pattern of presence of MTP joint synovial hypertrophy and forefoot bursal hypertrophy within the forefoot is variable in over half our participants.

**Table 7:** The presence of person specific(n) stable forefoot pathology over 12 months for the RA participants.

	MTP joint synovial hypertrophy		MTP joint erosion		Forefoot bursal hypertrophy	
	**Left**	**Right**	**Left**	**Right**	**Left**	**Right**

Stable pathology presence, lateral	10	7	81	70	37	35

Stable pathology presence, medial	10	10	0	0	1	4

Stable absence of pathology	28	26	2	2	4	4

Stable equal distribution of pathology	1	3	1	0	12	8

Total stable pathology	49	46	84	72	54	51

Percentage of cohort	43%	40%	74%	63%	47%	45%

### Correlations of US detectable forefoot pathology and peak forefoot pressure values

Following the trend observations, the data was explored further to determine any significant associations between the presence of US detectable pathology and peak pressure values in each of the forefoot segments. Findings showed that there was a significant negative correlation between the presence of US detectable pathology and peak pressure values in the right foot lateral segment at follow up (PCC = -0.412, p = 0.046), but this only demonstrates borderline significance at the 5% level, and care should be taken when inferring from this level of evidence. No other significant associations were found in any of the other variables. The data was explored further to determine any significant associations between the changes of US detectable pathology and changes in peak pressure values. No other significant associations were detected.

## Discussion

This investigation is considered the first to identify the presence of soft tissue pathology within the forefoot using US and patterns of foot-shoe interface pressures in a large cohort of patients with RA at two time points. Primarily we have observed that peak plantar pressures measured at the foot-shoe interface, are most likely to occur in the medial aspect of the forefoot (confirmed at both time points). By contrast, US detectable soft tissue pathology, forefoot bursal hypertrophy (confirmed at both time points) and MTP joint synovial hypertrophy (confirmed at twelve months) are most likely to be present in the lateral aspect of the forefoot. Additionally, we have observed that in this patient group the location of US detectable forefoot soft tissue pathology and location of peak foot-shoe interface pressures vary substantially over time.

Our findings are thus important as it is our observation that, in clinical practice, the assessment of foot-shoe interface pressures for patients who have RA is increasing. In this patient group, clinical strategies to offload peak pressures over time may therefore require additional information, such as US imaging to prevent overloading of potential current soft tissue inflammation that may not be detected clinically [7,29,30].

Previously in RA participants peak plantar pressures have been investigated against radiological erosion scores. In a small group (N = 16) of RA participants with established disease (mean 13.0 years) significant associations between erosion scores in the lateral MTP joints (3^rd ^to 5^th^) and peak pressure under 3^rd ^to 5^th ^MTP joints were reported [15]. Others categorised RA participants with established disease, but in remission, (N = 50), into high and low forefoot erosion scores and found significantly higher forefoot peak pressures occurring in the high erosion group [11]. The latter authors also reported that the highest pressure values were under the 5^th ^MTP joint [11]. Interestingly, in a larger group (N = 62) of RA participants, with established disease (mean 8 years) and high frequency of foot symptoms (89%), significant associations were found between erosion scores and peak pressures at MTP joints 1 and 4 [13]. It is however difficult to directly compare these results with our findings as each investigation used barefoot pedobarographic systems to record foot pressures, used different erosion scores and all were cross sectional.

The presence of forefoot erosion is indicative of prevalent foot disease and in the present study erosions were predominantly evident within the lateral aspect of the forefoot. It was also found that half of our participants had stable unchanging MTP joint synovial hypertrophy and forefoot bursal hypertrophy. By contrast, for half of the cohort we found that the presence in status of MTP joint synovial hypertrophy and forefoot bursal hypertrophy varied substantially over the twelve month period. We have previously hypothesised that this indicates the formation and regression of soft tissue pathology within the forefoot is a dynamic process that may be related to biomechanical adaptation [8].

Otter et al. [10] proposed that high plantar pressures observed in RA participants may be associated with a pain avoidance strategy related to off-loading the main site of inflammatory pain such as MTP joint synovial hypertrophy. In this study analysis, we found no difference in the location of peak plantar pressures between the RA and healthy participants although our results indicate that RA participants have significantly higher values of peak plantar pressures than healthy participants. The latter findings concur with those of other investigators [13,14,31]. Our evidence that, over time, changes in US detectable soft tissue pathology appear to be out of phase with changes in foot-shoe interface pressures both temporally and spatially does support the suggestion that these patients biomechanically adapt their gait away from forefoot pathology. However, we only found a negative association between the presence of US detectable pathology and peak pressure values in the right foot, lateral segment, at follow up which only demonstrated borderline significance. We also have to consider that peak pressures may not be the most clinically useful variable to focus on in this patient population. Further prospective research utilising investigation of other foot pressure variables, such as duration of peak pressure, pressure-time integrals and centre of pressure relative to forefoot soft tissue pathology and patient reported foot symptoms would be useful to determine optimal clinical assessment protocols.

It is inevitable that the heterogenous nature of the RA cohort in this study leads to the complexity of changes associated with foot status. Indeed the DAS-28 scores over the twelve month period indicate a high level of active disease within the cohort, although treatment appears stable. It was notable that disease status, measured by ESR, CRP, well-being and DAS-28, was also substantially variable over the 12 months. These findings are consistent with the well documented variability of RA disease over time [32-34].

Given the temporal variability of RA disease over time it is surprising that there is a lack of longitudinal data related to measurement of plantar foot pressures in RA. Mostly what is known is attributable to cross-sectional analytical data [10,13,24,35]. There is even less available evidence that investigates foot-shoe interface pressures over time with most attempts at longitudinal investigation in this population using barefoot pedobarographic assessment [12,13,15]. A possible explanation is that measurement of foot-shoe interface pressures is highly dependent on the shoe condition and therefore tight control over confounding factors such as footwear and activity is required for interventional studies. Inherent in this however is a disconnect in previous research findings and clinical utility of in-shoe foot pressure measurement within the RA population. The OMERACT (Outcome Measures in Rheumatology) framework incorporates truth, discrimination and feasibility as a filter to aid decisions as to the applicability of measures [36]. The literature on plantar foot pressure measurement in RA suggests that the methods may be feasible [2] however, their ability to discriminate in clinical practice has yet to be determined. Our aim was therefore to examine the forefoot pathology of patients who had RA at two cross sectional time points relevant to their usual daily activities/habits to mimic routine clinical practice assessments. It was not feasible to control for footwear over such a long period of time and in such a large population, which may be controversial.

As a response to this we performed additional analyses and an analysis of variance to assess the confounding influences of footwear. We found that the location of peak foot pressure remained predominantly medial in all footwear types. However, at twelve months, for the left foot only, those wearing unsuitable shoes had higher peak pressure values. In this study therefore, it is possible that for the twelve month left foot data peak pressures for the whole group may be inflated.

There are several strengths within this study that include a longitudinal cohort follow up design, the large sample size, and that it was a pragmatic clinical study representative of secondary care in the UK. A few potential limitations should also be considered. Primarily our sample of participants was heterogenous, with established disease and treated within secondary care and thus may not be generalizable to all patients with RA. Another potential limitation within this study is that we did not include tenosynovitis within our US observations of the forefoot and were unable to delineate the soft tissue structures in detail. Arguably this approach may underestimate the presence of soft tissue pathology within the forefoot. Such a limitation may be rectified using Magnetic Resonance Imaging to delineate the soft tissue structures.

Finally, for pragmatic analysis we categorised the forefoot nominally into either medial or lateral segments and also amalgamated the foot pathology data due to low counts in these categories, thus caution is required in interpreting this observational data. No further statistical inferences could be made from this current analysis and we recommend that future work in this area would be of value, particularly in the use of US detectable foot pathology and interface foot-shoe pressure pattern identification and cluster analysis [37]. Ideally, future work could focus on whether it is the impact of discrete pathology, such as IM or SM bursal hypertrophy, MTP joint synovial hypertrophy or tenosynovitis that may be associated with high plantar forefoot pressures or whether there is an 'optimal level' of forefoot pathology that impacts on plantar foot pressures.

## Conclusion

We have observed that there are considerable variations in the presence and location of US detectable soft tissue forefoot pathology and patterns of foot-shoe interface pressures over time in a large cohort of participants who have RA. We also noted that, in patients with RA, the changes in US detectable soft tissue forefoot pathology may be out of phase with the location and values of peak interface foot-shoe pressures. This implies that, in this patient group, clinical strategies to offload observed peak pressures, measured at the foot-shoe interface, over time may require additional information, such as US imaging to prevent overloading of existent soft tissue inflammation.

## Competing interests

No benefits in any form have been received or will be received from a commercial party related directly or indirectly to the subject of this article.

## Authors' contributions

CB conceived of the study, carried out the ultrasound, foot pressure measurements and clinical foot assessments for the patients with RA, ultrasound assessments for the healthy control participants and drafted the manuscript. DC participated in the design of the study and helped to draft the manuscript. RA and JB participated in the design and recruitment of the control study participants and carried out the foot pressure measurements on the control participants and helped to draft the manuscript. AG conducted the repeated measures test on the FScan sensors and participated in the acquisition of foot pressure measurements for the RA participants. LH participated in the acquisition of foot pressure measurements for the RA participants and helped to draft the manuscript. JHB participated in the conception and design of the study and helped to draft the manuscript. CJE participated in the conception and design of the study and helped to draft the manuscript. NA participated in the conception and design of the study and helped to draft the manuscript. All authors read and approved the final manuscript.
